# Longitudinal MR imaging after unilateral MR-guided focused ultrasound thalamotomy: clinical and radiological correlation

**DOI:** 10.3389/fneur.2023.1272425

**Published:** 2023-10-06

**Authors:** Sarah E. Blitz, Melissa M. J. Chua, Patrick Ng, David J. Segar, Rohan Jha, Nathan J. McDannold, Matthew N. DeSalvo, John D. Rolston, G. Rees Cosgrove

**Affiliations:** ^1^Harvard Medical School, Boston, MA, United States; ^2^Department of Neurosurgery, Brigham and Women’s Hospital, Harvard Medical School, Boston, MA, United States; ^3^Department of Neurological Surgery, Keck School of Medicine, University of Southern California, Los Angeles, CA, United States; ^4^Department of Radiology, Brigham and Women’s Hospital, Harvard Medical School, Boston, MA, United States

**Keywords:** focused ultrasound, essential tremor, thalamotomy, thermal lesions, lesion persistence

## Abstract

**Introduction:**

Magnetic-resonance-guided focused ultrasound (MRgFUS) thalamotomy uses multiple converging high-energy ultrasonic beams to produce thermal lesions in the thalamus. Early postoperative MR imaging demonstrates the location and extent of the lesion, but there is no consensus on the utility or frequency of postoperative imaging. We aimed to evaluate the evolution of MRgFUS lesions and describe the incidence, predictors, and clinical effects of lesion persistence in a large patient cohort.

**Methods:**

A total of 215 unilateral MRgFUS thalamotomy procedures for essential tremor (ET) by a single surgeon were retrospectively analyzed. All patients had MR imaging 1 day postoperatively; 106 had imaging at 3 months and 32 had imaging at 1 year. Thin cut (2 mm) axial and coronal T2-weighted MRIs at these timepoints were analyzed visually on a binary scale for lesion presence and when visible, lesion volumes were measured. SWI and DWI sequences were also analyzed when available. Clinical outcomes including tremor scores and side effects were recorded at these same time points. We analyzed if patient characteristics (age, skull density ratio), preoperative tremor score, and sonication parameters influenced lesion evolution and if imaging characteristics correlated with clinical outcomes.

**Results:**

Visible lesions were present in all patients 1 day post- MRgFUS and measured 307.4 ± 128.7 mm^3^. At 3 months, residual lesions (excluding patients where lesions were not visible) were 83.6% smaller and detectable in only 54.7% of patients (*n* = 58). At 1 year, residual lesions were detected in 50.0% of patients (*n* = 16) and were 90.7% smaller than 24 h and 46.5% smaller than 3 months. Lesions were more frequently visible on SWI (100%, *n* = 17), DWI (*n* = 38, 97.4%) and ADC (*n* = 36, 92.3%). At 3 months, fewer treatment sonications, higher maximum power, and greater distance between individual sonications led to larger lesion volumes. Volume at 24 h did not predict if a lesion was visible later. Lesion visibility at 3 months predicted sensory side effects but was not correlated with tremor outcomes.

**Discussion:**

Overall, lesions are visible on T2-weighted MRI in about half of patients at both 3 months and 1 year post-MRgFUS thalamotomy. Certain sonication parameters significantly predicted persistent volume, but residual lesions did not correlate with tremor outcomes.

## 1. Introduction

Magnetic resonance-guided focused ultrasound (MRgFUS) thalamotomy has demonstrated successful treatment of medication-refractory tremor in essential tremor (ET) (1, 2). This technique uses transcranial acoustic energy to ablate the ventral intermediate (Vim) nucleus of the thalamus, allowing for an incisionless approach in an awake patient to relieve debilitating tremor. Clinical neurologic feedback between sonications can guide target placement and other parameters, such as number of sonications, maximum power, and maximum energy. Sonications continue until adequate tremor control is achieved, with the targeted tissue usually reaching a peak temperature of around 55–60°C (3).

**Figure 1:**
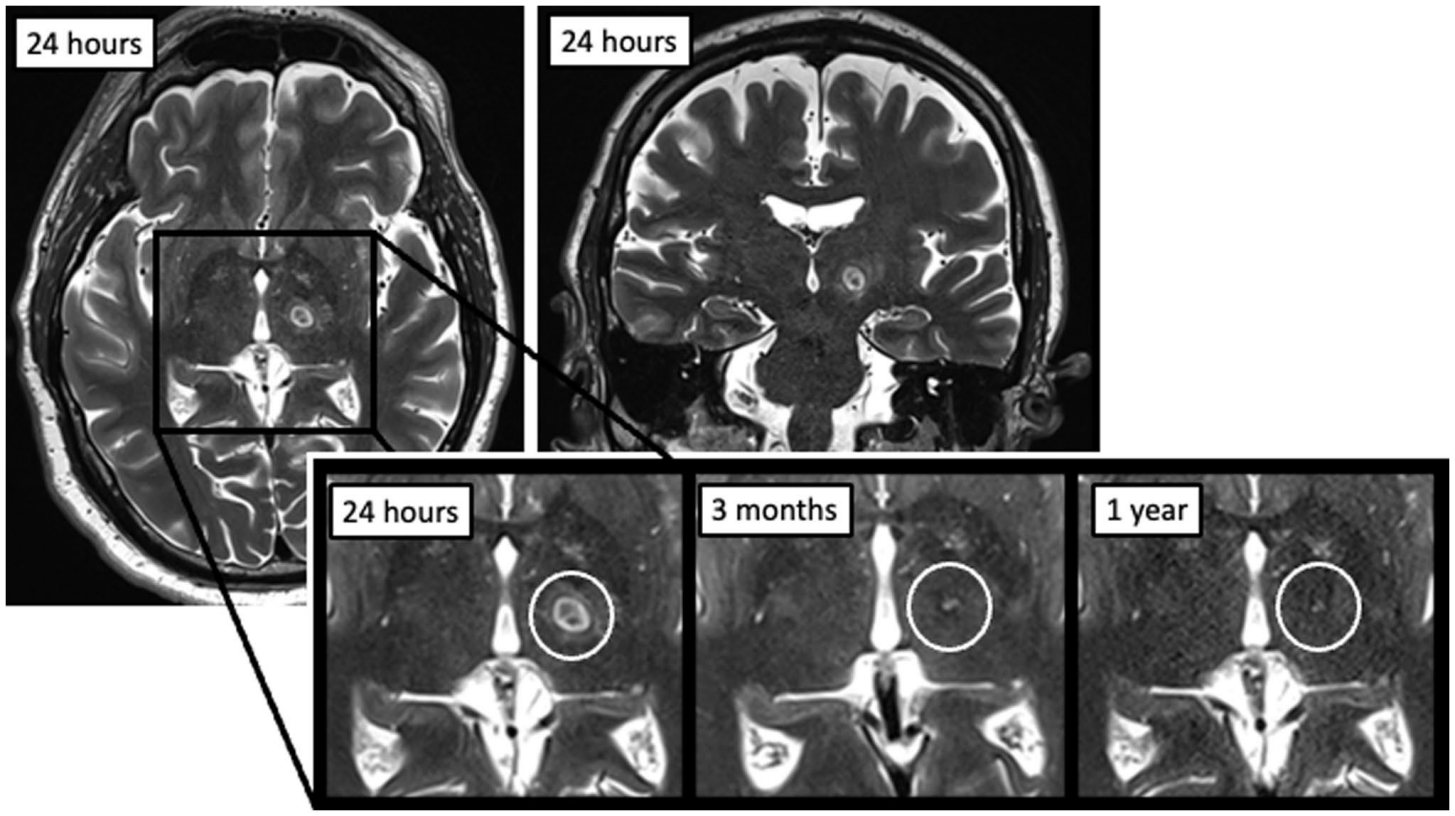
Example of an MRgFUS thalamotomy lesion at 24 h post-procedure on axial (left) and coronal (right) T2-weighted imaging, as well as evolution over time at three time points (24 h, 3 months, and 1 year).

Post-procedural imaging is common on the day after the procedure as well as at various time intervals thereafter. T2-weighted imaging 24-h after MRgFUS thalamotomy typically shows a region of ablation with dimensions of 6–8 mm, although specifics can vary depending on the exact time interval and MRI methods used (3). These lesions have distinct concentric zones: a central necrotic core (zone 1), surrounding cytotoxic edema (zone 2), and a larger ring of vasogenic edema (zone 3) (4). At our institution, additional MRI scans are often obtained at 3-months and less frequently at 1-year following the procedure depending on patient availability and ability to return to clinic. On these subsequent T2-weighted images, visible lesions are often substantially smaller or no longer visible despite persistent tremor improvement, which has previously been reported (4–7) (Figure. 1). There is currently no consensus on the frequency of postoperative imaging after MRgFUS thalamotomy, specific sequences to be used, or what these findings may represent. Some studies have looked at variables that correlate with post-procedure lesion persistence, but these have analyzed very few patients, mostly at less than 6 months post-procedure, and some only on two-dimensional axial imaging (4, 6, 8–10). Now that more bilateral thalamotomies are being performed, an understanding of lesion evolution over time will be useful to better assess how the second side target relates to the lesion location on the first side.

In this retrospective analysis of a large single-surgeon consecutive series of unilateral MRgFUS thalamotomy procedures in patients with ET, we aimed to evaluate the evolution and incidence of lesion persistence on T2-weighted MR imaging and correlate this with tremor outcomes and side effects at 3-months and 1-year. Prior studies have demonstrated that larger lesion size on MRI completed 1 day following MRgFUS thalamotomy is correlated with more adverse events (4, 11–13), and that lesion size and tremor control are influenced by various sonication parameters (10, 12, 14, 15). We therefore predicted that increasing sonication parameters (e.g., power, temperature, distance between individual sonications) would create larger lesions that would both persist on imaging as well as lead to more adverse effects, although potentially with improved tremor control.

## 2. Methods

### 2.1. Patient selection

We retrospectively identified all patients who underwent unilateral MRgFUS thalamotomy for ET between June 2017 and April 2022 at our institution. Those chosen for treatment included patients that satisfied the following criteria: (1) severe and/or disabling tremor, (2) failed multiple medications, (3) not a candidate for or unwilling to undergo deep brain stimulation (DBS), and (4) skull-density ratio of at least 0.35. All patients had imaging on postoperative day 1. Out of the 215 total patients treated, 121 had imaging at either 3 months, 1 year, or both timepoints following the procedure and were therefore included in the study.

### 2.2. MRgFUS procedure

The detailed MRgFUS thalamotomy procedural workflow at our institution has previously been published (11). Briefly, a patient’s head was completely shaved and a modified Codman-Robert-Wells frame (Radionics, Inc.) was applied under local anesthesia. A silicone membrane was stretched over the frame and head before placing the patient in a 3 T MRI (GE Medical Systems) connected to the ExAblate 4,000 MRgFUS transducer (InSightec Inc., Israel). The space between the transducer and the scalp was filled with cooled and degassed water for acoustic coupling. The unilateral Vim was targeted in all patients as previously described (8) using atlas-based coordinates of one quarter of the anterior commissure-posterior commissure (AC-PC) distance anterior to PC, 13–14 mm lateral to midline, and 1.5–2 mm superior to the midcommissural plane, and further refined with direct visualization (16). Subclinical test sonications were used to assess for transient tremor improvement or side effects and target adjustments were made as needed before delivery of high-powered sonications with maximum temperature of around 55–60°C (monitored with real-time MR thermometry). Patients were clinically assessed between each sonication to ensure adequate tremor control and monitor for side effects, including asking about sensory disturbances.

The postoperative focused ultrasound imaging protocol is as follows: 3D plane localizer sagittal and axial T1, axial 3D T1, axial and coronal 2 mm thin-cut T2, axial T2 GRE, axial Flair, axial SWI, and axial DWI. Later patients replaced SWI with WMn MPRAGE sequence on Prisma scanner and replaced DWI with DTI 30 direction (or 18 if 30 was not available).

### 2.3. Data collection and outcomes

For each procedure, sonication parameters were recorded including the total number of sonications, number of treatment sonications (defined as energy >5,000 J), maximum power, maximum energy, maximum duration, and maximum temperature. Additionally, with adjustment of the sonication target during the procedure to maximally relieve tremor, we recorded the maximum distance between planned targets. Skull-density ratio (SDR), or the ratio of cortical to cancellous bone, was calculated based on preoperative CT imaging. Because SDR is correlated with most sonication parameters given its impact on the required thermal dose to create lesions (10), SDR-normalized values of power, energy, and duration were calculated using a linear regression between the SDR and the respective sonication parameter, and fitting to a linear equation, as previously described (11). For each patient, SDR values were used to calculate the expected value based on the regression equation. Power, energy, or duration values for that patient were divided by the expected value, such that a value >1 corresponded to a value above the expected maximum. We also calculated the number of ‘low-power’ and ‘low-temperature’ sonications per treatment, since it was previously described that delivery of high power and temperature earlier in the treatment course led to larger lesions at 24 h (11). Because high-power sonications were previously defined as those within the top 10^th^ percentile, low-power sonications were defined as those within the bottom 90th percentile. This was also the definition used for low temperature sonications.

**Figure 2:**
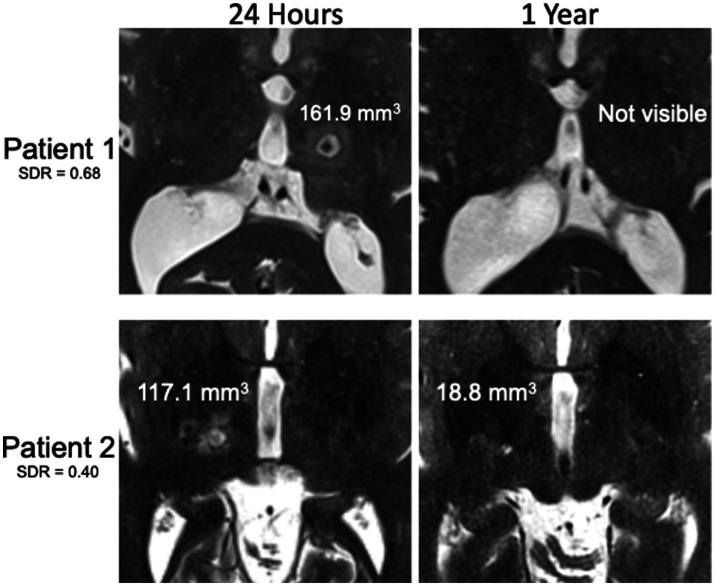
Examples of T2-weighted MR images 24 h and 1 year after MRgFUS for essential tremor. Patient with high SDR without visible lesion at 1 year despite large lesion at 24 h (top) compared to patient with low SDR with visible lesion at 1 year despite smaller lesion at 24 h (bottom). Lesion volumes displayed on the images.

Postoperative axial and coronal thin-cut (2 mm) T2-weighted images at 3 months and 1 year were analyzed on a binary scale for lesion visibility (authors SEB, MMJC, and DJS). This was done by comparing side-by-side images to the 24-h lesion and directly visualizing if there was any T2 hyper-or hypointense remnant in the location of the lesion. Figure. 2 shows an example of a patient with a lesion that was not present at 1 year (top) compared to a patient with a T2-bright lesion still present at 1 year (bottom). Approximate volumes were calculated for those with lesions still visible at 3 months and 1 year and for all 24-h lesions by measuring the anterior–posterior (AP), transverse (TR), and craniocaudal (CC) diameters calculating the volume of an ellipsoid, where *V* = volume of an ellipsoid, *a* = AP radius, *b* = TR radius, and *c* = CC radius:


$$V=\frac{4}{3}\mathrm{\pi abc}$$

At 24 h, 3 months, and 1 year post-MRgFUS thalamotomy, absolute volume as well as percent of 24-h volume still present were used for calculations.

Fahn-Tolosa-Marin (FTM) tremor scores were assessed preoperatively for baseline comparison, and at 3-month and 1-year postoperative follow-up visits. Tremor on the FTM scale is graded from 0 to 4, with 0 being no tremor, and 4 being severe tremor. FTM components assessed in each patient included head, voice, resting, postural, and intention tremor. Side effects were also recorded, including motor weakness, sensory deficits, dysarthria, fatigue, imbalance, dysgeusia, and coordination issues/dysmetria.

To assess attrition bias for patients with MRI at 1 year, the included patients were separated into 2 groups based on those that had a 1-year MRI versus those that did not. These groups were compared for patient characteristics, total and treatment sonications, volume at 24 h, preoperative total FTM score and FTM intention score, 1-year total FTM and FTM intention score, 1-year total FTM improvement, and 1-year FTM intention improvement.

### 2.4. Statistical analysis

Chi-squared tests, unpaired t-tests, and Spearman’s rank correlations were performed using Python version 3. When calculating Spearman’s rank correlations for volume, lesions that were not visible (i.e., volume of 0) were excluded given that the lesions could have become absent at different time points, which could impact the fit of the data. Multiple regressions were also used to determine predictors of lesion absence/presence as well as volume at these timepoints and change in volume at these timepoints. Variables included in the regression were based on previously determined significant predictors of volume at 24 h (11) as well as those found to be significant in simple Spearman’s rank correlations. A value of p of less than 0.05 was considered statistically significant. A Bonferroni test was used for post-hoc multiple-comparison correction.

## 3. Results

### 3.1. Patient baseline characteristics

Patients’ ages ranged from 59 to 94 years (mean ± SD, 75.4 ± 6.7), with most patients being male (71.1%) (Table 1). Baseline preoperative FTM scores ranged from 3 to 16 (7.0 ± 2.4) with FTM intention score ranging from 1 to 4 (3.2 ± 0.7).

**Table 1:** Patient characteristics.

	*n* = 121
Age, years, mean ± SD (range)	75.4 ± 6.7 (59–94)
Male sex, *n* (%)	86 (71.1)
Dominant hand, *n* (%)	
Left	20 (16.5)
Right	97 (80.2)
Ambidextrous	5 (4.1)
Right hand treatment, *n* (%)	96 (79.3)
Baseline preoperative FTM, median (IQR), range	7 (5–8), 3–16
Baseline preoperative FTM intention, median (IQR), range	3 (3–4), 1–4
3-month total FTM, median (IQR), range	0 (0–1), 0–12
3-month FTM intention, median (IQR), range	0 (0–0), 0–4
3-month FTM intention percent improvement, median (IQR), range	100.0 (100.0–100.0), 0.0–100.0
1-year total FTM, median (IQR), range	0 (0–1), 0–9
1-year FTM intention, median (IQR), range	0 (0–1), 0–4
1-year FTM intention percent improvement, mean ± SD	100.0 (75.0–100.0), 0.0–100.0

### 3.2. Lesion visibility and volume

**Figure 3:**
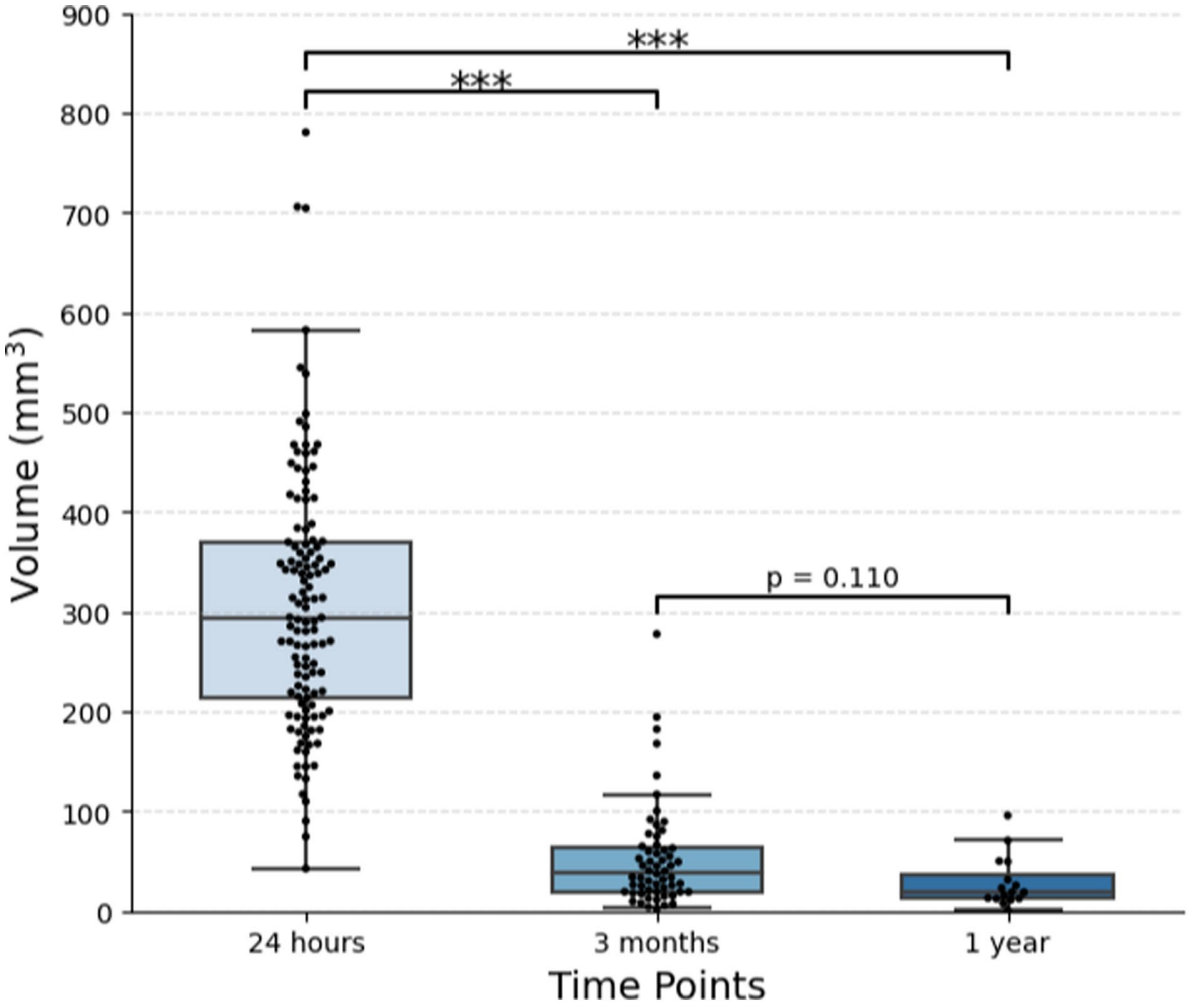
Change in lesion volume over time. One hundred and twenty-one patients with essential tremor who underwent MRgFUS thalamotomy were included. Out of 106 patients with imaging at 3 months, 58 had visible lesions. Out of 32 patients with imaging at 1 year, 16 had visible lesions. The plot displays the median and IQR (***p < 0.001).

All patients had visible lesions on 24-h post-procedural T2-weighted MRI, with an average volume of 307.4 ± 128.7 mm^3^ [median (IQR), 294.2 (213.9–369.9) mm^3^], or 7.7 × 7.9 × 9.2 mm (AP x TR x CC) (Table 2; Figures 1, 3). At 3 months, residual lesions were detectable in 59.0% of patients (*n* = 69) and were 83.6% smaller on average with a volume of 38.7 (19.2–64.4) mm^3^ (*p* < 0.0001), or 3.9 × 4.7 × 4.5 mm. For patients who had SWI sequences (*n* = 17), visible SWI signal was present in all at 3 months. The majority of lesions were also visible on DWI (*n* = 38, 97.4%) and ADC (*n* = 36, 92.3%) at 3 months. Almost half were bright on DWI (42.1%). Lesions at 1 year were detectable in 52.9% of patients (*n* = 18). Residual lesions [18.9 (12.5–35.9) mm^3^ or 3.1 × 3.9 × 3.9 mm] were on average 90.7% smaller than 24 h (*p* < 0.0001), and 46.5% smaller than 3 months (*p* = 0.110).

**Table 2:** Lesion characteristics at 3 months and 1 year postoperatively on MRI.

**24 h**		
T2-weighted lesion presence	*n* = 121 (100%)	
Volume, median (IQR)	294.2 (213.9–369.9) mm^3^	

**3 months**		*p* value (compared to 24 h)
T2-weighted lesion presence	*n* = 58 (54.7%)	**<0.0001**
Volume, median (IQR)	38.7 (19.2–64.4) mm^3^	**<0.0001**
SWI lesion presence	*n* = 17 (100%)	
DWI lesion presence	*n* = 38 (97.4%)	
Bright on DWI	*n* = 16 (42.1%)	
ADC lesion presence	*n* = 36 (92.3%)	

**1 year**		*p* value (compared to 24 h)	*p* value (compared to 3 months)
T2-weighted lesion presence	*n* = 16 (50.0%)	**<0.0001**	0.393
Volume, median (IQR)	18.8 (12.5–35.9) mm^3^	**<0.0001**	0.110

The percent of lesions present and volumes of lesions were significantly smaller at 3 months compared to 24 h and 1 year compared to 24 h.

### 3.3. Predictors of lesion visibility and size

Independent t-tests between patients with lesions present and those without lesions present at both 3 months and 1 year demonstrated no difference in patient characteristics (age and SDR), sonication parameters, preoperative tremor scores, or 24-h lesion volume (Supplementary Table S1).

**Figure 4:**
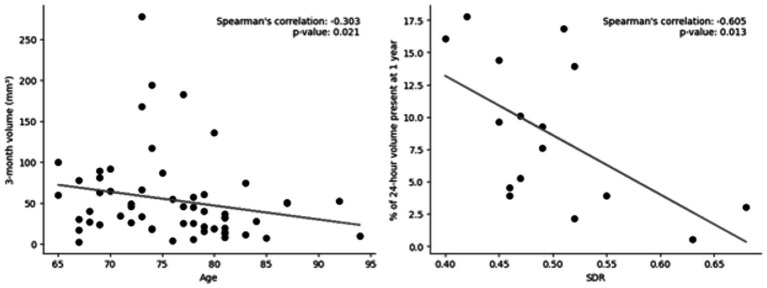
Scatter plots of predictors of volume at various timepoints. Left: Lesion volume at 3 months vs. age; out of 106 patients who had imaging at 3 months, 58 still had lesions present. Right: Skull density ration (SDR) vs. change in volume at 1 year; Out of 32 patients who had imaging at 1 year, 16 still had lesions present. Linear trendline and Spearman’s rank correlation value and value of p are included. Neither were significant after Bonferroni correction (*p* < 0.003).

Before multiple-comparison corrections, Spearman’s rank correlations found that age predicted the lesion volume at 3 months (rho = −0.302, *p* = 0.021) and SDR predicted the change in volume at 1 year (rho = −0.605, *p* = 0.013) (Figure 4; Supplementary Table S1). However, after Bonferroni corrections, neither of these remained significant (not *p* < 0.003).

Multiple regressions to determine predictors of lesion presence at both 3 months and 1 year did not show any significant variables. A regression of predictors of lesion volume at 3 months showed that the number of treatment sonications (coefficient = −12.7, *p* = 0.016), normalized maximum power (coefficient = 96.5, *p* = 0.048), and the maximum distance between sonication targets (coefficient = 27.9, *p* = 0.013) predicted volume. Treatment sonications and maximum distance also predicted the percent of lesion volume still present at 3 months compared to 24-h volume (coefficient = −5.7, *p* = 0.003 and coefficient = 10.5, *p* = 0.009, respectively). No variables were significant in the 1-year volume regressions.

### 3.4. Lesion visibility and volume and outcomes

At 3 months, the total FTM score ranged from 0 to 12 [median (IQR) = 0 (0–1)], and FTM intention ranged from 0 to 4 [0 (0–0)]. At 1 year, the total FTM score ranged from 0 to 9 [0 (0–1)], and FTM intention ranged from 0–4 [0 (0–1)]. Lesion visibility and lesion size at 3 months and 1 year did correlate with some side effects, specifically weakness and sensory deficits (Supplementary Table S3). After Bonferroni corrections, the only significant correlate was that those with sensory deficits at 3 months had larger lesions (*p* = 0.001). Interestingly, tremor outcomes were not found to be related to the presence of the lesion or lesion size.

### 3.5. Assessing attrition bias at 1 year

Patients who had MRIs at 1 year post-MRgFUS thalamotomy (*n* = 34), compared to those who did not (*n* = 87), were significantly younger (73.2 ± 6.8 vs. 76.3 ± 6.5, *p* = 0.019). There was similar gender distribution (*p* = 0.603) and SDRs (*p* = 0.489). However, those who did have MRIs underwent significantly more total sonications (6 ± 3 vs. 5 ± 2, *p* = 0.015) and treatment sonications (11 ± 5 vs. 9 ± 3, *p* = 0.002). Additionally, patients who did have MRIs were significantly earlier patients in the cohort with lower identification numbers (*p* < 0.001). Preoperative total FTM scores and FTM intention scores were not significantly different (*p* = 0.703 and *p* = 0.715, respectively). Lesion volumes at 24 h were also similar (*p* = 0.427). At 1 year, FTM intention scores and percent improvement were not significantly different (*p* = 0.317 and *p* = 0.356, respectively).

## 4. Discussion

There is no current consensus on the utility or frequency of postoperative imaging after unilateral MRgFUS thalamotomy. In this single-surgeon series, we show that lesions are visually identifiable on T2-weighted MRI in only about one half of patients at both 3 months and 1 year following MRgFUS thalamotomy (Table 2). This may be slightly higher than would be seen in other cohorts, given our institution’s larger 24-h lesions (11). Keil et al. describe similar findings of lesion persistence on T2-weighted imaging in only 40% of their patients at 6 months postoperatively with smaller lesion volumes at all timepoints post-procedurally (3 days, 1 month, and 6 months) (6). These observations demonstrate that while T2-weighted images are very useful during the first few days after MRgFUS to show lesion location, extent and edema, the images may return to normal after 1 month and are inadequate for longer follow-up studies (4, 6). SWI, on the other hand, demonstrates persistent lesions more reliably over long-term follow-up (Table 2) (6). This suggests that SWI could be used in postoperative imaging protocols to localize lesions after extended periods of time, especially when planning retreatments or contralateral treatments.

### 4.1. Significant predictors of lesion persistence

#### 4.1.1. Some sonication parameters predicted 3-month volume

The multivariate regression for lesion size at 3 months showed that the number of treatment sonications, maximum power, and maximum distance between sonication targets predicted lesion size. A fewer number of treatment sonications was correlated with increased lesion size as well as a larger percentage of lesion volume still present at 3 months confirming what we have previously found: more, lower power sonications does not create as large of a lesion as fewer, higher power sonications (11). Here, we show that this finding seems to persist on imaging past 24 h. Greater maximum power and maximum distance positively predicted lesion volume, which has also previously been shown on 24-h imaging (11). Overall, these known predictors of larger 24-h volume also predict larger 3-month volume.

#### 4.1.2. Increased SDR may lead to greater change in volume at 1 year

Although not significant after post-hoc corrections or within the multivariate regression, when assessed independently, patients with lower SDR had more of their lesion still present at 1 year (Figure 4; Supplementary Table S2). This was not seen at 3 months. Heating efficiency is known to be worse for patients with a lower SDR (17–20). It has previously been demonstrated that the skull along the ultrasound beam paths can cause acoustic parameters to change, leading to blurring (dephasing) of the focus and reduction in treatment efficiency (21). Greater heating efficiency for patients with high SDR may not have the same impact on the tissue, leading to lesions that do not maintain the same volume. At lower SDRs, the ultrasound focus disperses, leading to less precise targeting (21). The impact on the tissue surrounding the lesion may be impacted in a way that maintains the structure of the lesion without collapsing in on itself as quickly at this long-term follow-up.

The multivariate regression at 1 year showed no significant predictors. This is likely because there were very few observations (only 32 patients with 1-year imaging, and only 16 still had lesions present), which would not allow for any smaller associations to be extrapolated.

### 4.2. Clinical outcomes

#### 4.2.1. Radiological persistence of lesion does not correlate with tremor outcomes

We did not find any correlations between presence or volumes of lesions on 3-months or 1-year T2-weighted MRI with any metrics of tremor improvement (Supplementary Table S1). This may also reflect, as we have suggested previously (11), that lesions at our institution are larger than those reported by several other series, and that even our smaller lesions tend to remain above any threshold that might correlate with changes in tremor improvement. Although lesion size on 1 day postoperative imaging has been shown to predict tremor outcomes up to 1 year after MRgFUS thalamotomy (4, 11, 12), studies have repeatedly demonstrated that tremor control persists despite lesions disappearing on T2-weighted imaging (4–7). Unlike our results, Keil et al. found that greater lesion shrinkage at 180 days on T2-weighted MRI correlated with increased tremor recurrence on the treated limb (6). It is possible that with additional patients or with imaging at additional timepoints, we too may have seen correlation with tremor outcomes. Additionally, as previously discussed, other imaging sequences, such as SWI where lesions are present for longer, may provide better insight into this question.

#### 4.2.2. Radiological evidence and volume of lesion predicts side effects

After post-hoc analysis, patients with sensory side effects at 3 months had larger lesions (Supplementary Table S3). This was not surprising, given that sensory side effects are very common and related to larger or more posterior lesions at 24-h that overlap with the ventroposterolateral nucleus which lies posterior to the Vim (11, 22). While previous studies have found that larger lesions on 24-h imaging is correlated with more adverse effects (11), to the best of our knowledge, this is the first time that postoperative imaging beyond the first few days has demonstrated a significant correlation with adverse events.

### 4.3. Limitations and future directions

While we describe a very large cohort with imaging at 24 h after MRgFUS thalamotomy (*n* = 121), this number decreased for those with imaging and FTM scores at 3 months (*n* = 99) and at 1 year (*n* = 28). Analyses with more patients could gain greater statistical power and may elucidate more subtle correlations. Additionally, there were some differences between the patient population that had MRIs at 1 year compared to those that did not, including imaged patients being younger and having undergone more total and treatment sonications. Future studies with more consistent imaging could avoid attrition bias. Another limitation is that the determination of presence or absence of lesions has intrinsic subjectivity which we attempted to mitigate by having a second observer where necessary. Finally, due to the imaging preferences at our institution, SWI sequences were not obtained for many patients, which did not allow for any significant analysis outside of noting presence for all images.

An interesting future direction is analyzing diffusion tensor imaging (DTI) over time. Previous studies have demonstrated persistent changes in tractography up to 1 year after MRgFUS thalamotomy (23). This type of imaging gives much clearer information into the specific fiber tracts that are targeted during FUS procedures to elicit the intended effects. Various features of connectivity have been correlated with better tremor outcomes (24–31). With longitudinal studies, DTI may give insight into the individual changes in functional connectivity of tremor circuitry that lead to persistent adverse effects as well as tremor control and/or recurrence.

## 5. Conclusion

Lesions are visible on T2-weighted MRI in only about one half of patients at both 3 months and 1 year post unilateral MRgFUS thalamotomy for ET and were significantly smaller over time. At 3 months, multivariate regression showed that fewer treatment sonications, greater maximum power, and larger distance between treatment sonication targets led to more persistent lesions. Independent analyses showed that older patients tended to have smaller 3-month volumes. While the presence or size of lesion at 3 months was not a predictor of tremor outcomes, it did predict sensory side effects. Overall, postoperative T2-weighted MR imaging at 3 months and beyond did not provide significant clinically relevant data and SWI MRI sequences may be better to analyze predictors and significance in volume changes. Moving forward, as we start to perform more bilateral FUS procedures, this study supports relying on early postoperative imaging for procedure planning.

## Data availability statement

The raw data supporting the conclusions of this article will be made available by the authors, without undue reservation.

## Ethics statement

Ethical approval was not required for the studies involving humans because it was a retrospective review of unidentifiable data. The studies were conducted in accordance with the local legislation and institutional requirements. Written informed consent for participation was not required from the participants or the participants’ legal guardians/next of kin in accordance with the national legislation and institutional requirements because it was a retrospective review of unidentifiable data.

## Author contributions

SB: Data curation, Formal analysis, Investigation, Methodology, Software, Visualization, Writing – original draft. MC: Data curation, Investigation, Methodology, Writing – review & editing. PN: Data curation, Writing – review & editing. DS: Formal analysis, Writing – review & editing. RJ: Data curation, Writing – review & editing. NM: Data curation, Writing – review & editing. MD: Data curation, Writing – review & editing. JR: Supervision, Writing – review & editing. GC: Conceptualization, Supervision, Writing – review & editing.
