# Supplementary material for: Longitudinal MR imaging after unilateral MR-guided focused ultrasound thalamotomy: clinical and radiological correlation
Source: Front Neurol. 2023 Oct 6;14:1272425. doi: 10.3389/fneur.2023.1272425 (PMC10587555; doi:10.3389/fneur.2023.1272425)
Supplement: Supplementary file 1 [file Table_1.docx]

Supplementary Material

# Supplementary Tables

**Supplemental Table 1.** Assessment of characteristics’ correlation with T2-weighted lesion presence.

| **3 months** | **Lesion presence**  **(mean ± SD)** | **Lesion absence**  **(mean ± SD)** | ***p* value** |
| --- | --- | --- | --- |
| **Characteristics** | **n = 58** | **n = 48** |  |
| Age | 76.1 ± 6.5 | 75.8 ± 6.2 | 0.789 |
| SDR | 0.50 ± 0.08 | 0.48 ± 0.08 | 0.242 |
| **Sonication parameters** | **n = 58** | **n = 47** |  |
| Total sonications | 9 ± 3 | 8 ± 2 | 0.061 |
| Treatment sonications | 5 ± 3 | 4 ± 2 | 0.130 |
| Number of low-power sonications | 8 ± 2 | 7 ± 2 | 0.334 |
| Number of low-temperature sonications | 6 ± 3 | 5 ± 2 | 0.089 |
| Normalized maximum power | 1.00 ± 0.22 | 0.99 ± 0.14 | 0.906 |
| Normalized maximum energy | 1.04 ± 0.67 | 1.09 ± 0.43 | 0.692 |
| Normalized maximum duration | 0.98 ± 0.33 | 1.06 ± 0.37 | 0.248 |
| Maximum temperature (^o^C) | 62.0 ± 3.9 | 61.9 ± 3.6 | 0.922 |
| Maximum distance between targets (mm) | 1.4 ± 1.0 | 1.1 ± 0.7 | 0.086 |
| **Preoperative FTM score** | **n = 58** | **n = 48** |  |
| Total FTM score | 7.3 ± 2.8 | 6.5 ± 1.9 | 0.143 |
| FTM intention score | 3.3 ± 0.7 | 3.1 ± 0.8 | 0.210 |
| 24-hour volume | 310.8 ± 136.0 | 303.7 ± 100.6 | 0.766 |
|  |  |  |  |
| **1 year** |  |  |  |
| **Characteristics** | **n = 16** | **n = 16** |  |
| Age | 70.8 ± 5.5 | 75.0 ± 7.1 | 0.075 |
| SDR | 0.50 ± 0.07 | 0.49 ± 0.08 | 0.676 |
| **Sonication parameters** | **n = 16** | **n = 16** |  |
| Total sonications | 12 ± 6 | 10 ± 4 | 0.161 |
| Treatment sonications | 7 ± 4 | 6 ± 2 | 0.450 |
| Number of low-power sonications | 11 ± 6 | 9 ± 4 | 0.314 |
| Number of low-temperature sonications | 9 ± 6 | 6 ± 4 | 0.132 |
| Normalized maximum power | 1.08 ± 0.19 | 1.04 ± 0.14 | 0.571 |
| Normalized maximum energy | 1.01 ± 0.30 | 0.93 ± 0.40 | 0.526 |
| Normalized maximum duration | 0.93 ± 0.26 | 0.84 ± 0.33 | 0.436 |
| Maximum temperature (^o^C) | 61.2 ± 3.2 | 62.8 ± 3.5 | 0.205 |
| Maximum distance between targets (mm) | 1.4 ± 0.7 | 1.1 ± 0.6 | 0.342 |
| **Preoperative FTM score** | **n = 16** | **n = 16** |  |
| Total FTM score | 7.7 ± 3.0 | 6.4 ± 1.5 | 0.161 |
| FTM intention score | 3.4 ± 0.6 | 3.1 ± 0.6 | 0.149 |
| 24-hour volume | 325.7 ± 134.0 | 288.4 ± 120.1 | 0.429 |

**Supplemental Table 2**. *p* values and Spearman correlation coefficients of Spearman’s Correlation, accounting for Record ID, of variables in relation to lesion volume at 24 hours, 3 months, and 1 year post procedure as well as percent decrease in volume (Δ volume) compared to 24-hour volume.

| p value (correlation coefficient) | **3-month volume** | **3-month**  **Δ volume** | **1-year volume** | **1-year**  **Δ volume** |
| --- | --- | --- | --- | --- |
| **Patient characteristics** |  |  |  |  |
| Age | **0.021 (-0.303)** | 0.416 (-0.109) | 0.172 (0.359) | 0.372 (0.239) |
| SDR | 0.742 (-0.044) | 0.080 (-0.231) | 0.092 (-0.435) | **0.013 (-0.605)** |
| **Sonication parameters** |  |  |  |  |
| Total sonications | 0.580 (-0.074) | 0.637 (-0.063) | 0.106 (-0.419) | 0.978 (-0.007) |
| Treatment sonications | 0.206 (-0.169) | 0.254 (-0.152) | 0.177 (-0.355) | 0.930 (-0.024) |
| Number of low-power sonications | 0.852 (-0.025) | 0.622 (0.066) | 0.747 (-0.088) | 0.268 (0.294) |
| Number of low-temperature sonications | 0.651 (-0.061) | 0.595 (0.071) | 0.111 (-0.414) | 0.935 (-0.022) |
| Normalized maximum power | 0.408 (0.111) | 0.943 (0.010) | 0.431 (-0.212) | 0.345 (-0.253) |
| Normalized maximum energy | 0.722 (-0.048) | 0.970 (0.005) | 0.196 (-0.341) | 0.169 (-0.0362) |
| Normalized maximum duration | 0.451 (-0.101) | 0.795 (0.035) | 0.549 (-0.162) | 0.688 (-0.109) |
| Maximum temperature | 0.694 (0.053) | 0.106 (-0.215) | 0.145 (0.381) | 0.812 (0.065) |
| Maximum distance between targets | 0.484 (0.094) | 0.890 (0.019) | 0.095 (0.431) | 0.386 (0.233) |
| **Tremor** |  |  |  |  |
| Preoperative total FTM | 0.672 (-0.057) | 0.815 (0.031) | 0.312 (0.270) | 0.514 (0.176) |
| Preoperative FTM intention | 0.817 (0.031) | 0.677 (0.056) | 0.169 (0.361) | 0.119 (0.406) |
| FTM intention improvement (%) | 0.197 (0.178) | 0.715 (0.051) | 0.304 (0.324) | 0.841 (-0.065) |
| 24-hour volume | 0.069 (0.240) | 0.134 (-0.199) | 0.068 (0.468) | 0.948 (0.018) |

**Supplemental Table 3.** Outcomes of T2-weighted lesion presence and volume.

| **3 Months** | **Lesion presence**  (n = 53) | **Lesion absence**  (n = 46) | ***p* value** | **Side effect present** | **Side effect absent** | ***p* value** |
| --- | --- | --- | --- | --- | --- | --- |
| **Side Effects, n (%)** |  |  |  | **Volume**  **(median [IQR]) (mm^3^)** | **Volume**  **(median [IQR]) (mm^3^)** |  |
| Any side effect | 36 (67.9) | 20 (43.5) | **0.025** | 45.6 (24.7 – 77.8) | 30.9 (15.6 – 55.6) | **0.044** |
| Fatigue | 4 (7.5) | 3 (6.5) | 1.0 | 32.1 (23.4 – 73.5) | 40.1 (19.3 – 63.0) | 0.675 |
| Dysarthria | 3 (5.6) | 1 (2.2) | 0.714 | 50.9 (33.1 – 164.3) | 34.0 (19.2 – 61.4) | **0.039** |
| Weakness | 5 (9.4) | 2 (4.3) | 0.554 | 135.8 (19.1 – 167.9) | 34.0 (19.3 – 57.6) | **0.023** |
| Sensory | 23 (37.7) | 11 (23.9) | 0.207 | 57.3 (37.0 – 98.1) | 28.6 (17.4 – 48.5) | **0.001** |
| Imbalance | 15 (28.3) | 8 (17.4) | 0.297 | 40.1 (24.5 – 63.9) | 34.0 (17.5 – 58.7) | 0.402 |
| Dysgeusia | 4 (7.5) | 5 (10.9) | 0.823 | 57.3 (46.4 – 74.0) | 33.7 (19.1 – 61.7) | 0.698 |
| Dysmetria/discoordination | 5 (9.4) | 2 (4.3) | 0.554 | 33.1 (27.0 – 49.3) | 37.2 (19.1 – 63.0) | 0.878 |
| **Tremor Outcomes, mean ± SD** | **FTM score** | **FTM score** |  |  |  |  |
| 3-month FTM score | 0.9 ± 2.3 | 0.5 ± 0.9 | 0.206 |  |  |  |
| 3-month FTM intention | 0.3 ± 0.9 | 0.3 ± 0.6 | 0.648 |  |  |  |
| FTM intention improvement (%) | 90.0 ± 24.6 | 91.7 ± 17.1 | 0.698 |  |  |  |
|  |  |  |  |  |  |  |
| **One Year** | (n = 12) | (n = 16) |  |  |  |  |
| **Side Effects, n (%)** |  |  |  | **Volume**  **(median [IQR])**  **(mm^3^)** | **Volume**  **(median [IQR]) (mm^3^)** |  |
| Any side effect | 6 (50.0) | 8 (50.0) | 1.0 | 34.2 (14.1 – 50.0) | 17.2 (12.5 – 25.0) | 0.216 |
| Fatigue | 0 | 0 |  |  |  |  |
| Dysarthria | 0 | 0 |  |  |  |  |
| Weakness | 0 | 0 |  |  |  |  |
| Sensory | 3 (25.0) | 3 (18.8) | 1.0 | 50.1 (49.8 – 72.9) | 15.6 (12.3 – 18.8) | **0.011** |
| Imbalance | 3 (25.0) | 4 (25.0) | 1.0 | 12.5 (10.1 – 15.7) | 25.7 (15.6 – 50.1) | 0.213 |
| Dysgeusia | 1 (8.3) | 1 (6.3) | 1.0 | 49.6 | 18.8 (12.4 – 37.9) |  |
| Dysmetria/discoordination | 0 | 2 (12.5) | 0.596 |  |  |  |
| **Tremor Outcomes, mean ± SD** | **FTM score** | **FTM score** |  |  |  |  |
| 1-year FTM score | 0.3 ± 0.4 | 0.5 ± 0.5 | 0.193 |  |  |  |
| 1-year FTM intention | 0.2 ± 0.4 | 0.4 ± 0.5 | 0.243 |  |  |  |
| FTM intention improvement (%) | 95.8 ± 9.3 | 87.0 ± 17.4 | 0.139 |  |  |  |

*Numbers are slightly different than Supp Table 1 given that some patients had MRIs at these time points but did not have documented clinical outcomes.*
